# Ionizing radiation results in a mixture of cellular outcomes including mitotic catastrophe, senescence, methuosis, and iron-dependent cell death

**DOI:** 10.1038/s41419-020-03209-y

**Published:** 2020-11-23

**Authors:** Sandy Adjemian, Teodora Oltean, Sofie Martens, Bartosz Wiernicki, Vera Goossens, Tom Vanden Berghe, Benjamin Cappe, Maria Ladik, Franck B. Riquet, Liesbeth Heyndrickx, Jolien Bridelance, Marnik Vuylsteke, Katrien Vandecasteele, Peter Vandenabeele

**Affiliations:** 1 Unit of Molecular Signaling and Cell Death, VIB Center for Inflammation Research, Ghent, Belgium; 2 Department of Biomedical Molecular Biology, Cancer Research Institute Ghent (CRIG), Ghent University, Ghent, Belgium; 3 Cancer Research Institute Ghent (CRIG), Ghent, Belgium; 4 VIB Screening Core & UGhent Expertise Centre for Bioassay Development and Screening (C-BIOS), VIB, UGhent, Ghent, Belgium; 5 Laboratory of Pathophysiology, Department of Biomedical Sciences, University of Antwerp, Antwerp, Belgium; 6 Université de Lille, Lille, France; 7 Gnomixx, Melle, 9090 Belgium; 8 Department of Radiation Oncology and Experimental Cancer Research, Ghent University, Ghent, Belgium; 9 Radiation Oncology, Ghent University Hospital, Ghent, Belgium; 10 Methusalem program, Ghent University, Ghent, Belgium

**Keywords:** Radiotherapy, Cell death, Senescence

## Abstract

Radiotherapy is commonly used as a cytotoxic treatment of a wide variety of tumors. Interestingly, few case reports underlined its potential to induce immune-mediated abscopal effects, resulting in regression of metastases, distant from the irradiated site. These observations are rare, and apparently depend on the dose used, suggesting that dose-related cellular responses may be involved in the distant immunogenic responses. Ionizing radiation (IR) has been reported to elicit immunogenic apoptosis, necroptosis, mitotic catastrophe, and senescence. In order to link a cellular outcome with a particular dose of irradiation, we performed a systematic study in a panel of cell lines on the cellular responses at different doses of X-rays. Remarkably, we observed that all cell lines tested responded in a similar fashion to IR with characteristics of mitotic catastrophe, senescence, lipid peroxidation, and caspase activity. Iron chelators (but not Ferrostatin-1 or vitamin E) could prevent the formation of lipid peroxides and cell death induced by IR, suggesting a crucial role of iron-dependent cell death during high-dose irradiation. We also show that in K-Ras-mutated cells, IR can induce morphological features reminiscent of methuosis, a cell death modality that has been recently described following H-Ras or K-Ras mutation overexpression.

## Introduction

Radiotherapy is widely used for cancer treatment and although it can target both normal and cancerous cells, fractionated radiotherapy using repeatedly low doses per fraction limits damage to the normal tissue^1^. Therefore, a standard radiotherapy regimen using daily doses of around 2 Gy over 5–7 weeks is used in many tumor types (National Comprehensive Cancer Network Guidelines). Yet, several studies reported that repeated irradiation with a low dose can induce a chronic inflammation, while high doses boost the immune system^2,3^. Recent technological advances enable delivering high dose per fraction (in fewer fractions, i.e. hypofractionation) with minimal damage to the surrounding tissue. Nonetheless, further investigation is needed to shift the standard of repeated low dose to high-dose treatment.

Ionizing radiation (IR) induces direct damages to the DNA causing single- or double-strand breaks (SSBs, DSBs). Nevertheless, around 70% of the DNA damage is actually caused by free radicals generated following the ionization of water in the cells^4^. These events engage the DNA damage response (DDR) pathway, and while the SSBs are readily repaired, around 5% of DSBs fail to be repaired and thus ultimately lead to cell death^5^. Due to their higher replication rate and defects in the DDR pathway, cancer cells are more susceptible to succumb to IR as compared with normal cells. Following DNA damage, cell cycle checkpoints stop cycle progression preventing replication of damaged DNA. Depending on the cell cycle phase during IR, radiosensitivity and mechanisms leading to cell cycle arrest are different^6^. When DNA damage occurs prior or during mitosis, cells undergo mitotic catastrophe, and as a consequence become binucleated and are not able to complete their cycle^7^. Eventually, these cells may become senescent or die through apoptosis or necrosis^7,8^.

As mentioned above, IR generates free radicals^9^. Among them, reactive oxygen species (ROS, such as hydroxyl radical HO• or hydrogen peroxide H_2_O_2_) are produced from oxygen metabolism, are toxic at high levels and can damage DNA, lipids, and proteins^9,10^.

Many studies described several types of cell death following IR, such as apoptosis, necrosis, and more recently necroptosis, a RIPK3/MLKL regulated type of necrosis^11–13^. These cell death modalities have a distinct outcome on the immune system. While necrosis and necroptosis are more inflammatory^14,15^, apoptosis is considered a less immunogenic cell death modality. Several reviews suggested that while low doses IR would induce apoptosis, a higher dose would trigger necrosis^11,16^, suggesting that a low dose would be non-immunogenic, in contrast to a high dose.

The literature concerning IR-induced cell death is confusing, and results differ substantially depending on the cell line, or the dose. Systematic studies are lacking where different types of cells and IR doses are rigorously compared. We investigated the induction of regulated cell death mechanisms (apoptosis, necroptosis, ferroptosis) and cellular stress responses (mitotic catastrophe, senescence) by different doses of IR. We included a panel of murine cell lines of distinct origins (lung adenocarcinoma, cervical cancer, colon cancer, and fibrosarcoma). All the cell lines tested responded similarly to different doses of IR and exhibited dose-dependent caspase activity, features of mitotic catastrophe and senescence. Cancer cells also underwent lipid peroxidation that could be prevented by iron chelators (but not by free-radical traps^17,18^) that additionally inhibited IR-induced cell death, implying a crucial role for free iron-dependent cell death.

We also found that IR induced morphological features of methuosis in K-Ras-mutated cell lines (hereafter referred as methuosis). Methuosis is a cell death modality recently characterized occurring following expression of H-Ras or K-Ras mutation (G12V)^19,20^. It is characterized by massive vacuolization in the cytoplasm. These vacuoles are formed by macropinocytosis and instead of being normally recycled, fuse with each other. In a late phase of methuosis, cells detach and their membrane become permeabilized, without showing apoptotic features^21^.

Ras is frequently mutated in cancer, with K-Ras being the most prevalent mutation, present for example in 90% of pancreatic tumors^22^. In cancer patients, the presence of K-Ras mutations is frequently assessed and radiotherapy is often part of the proposed treatment^23^. Exploring the role of methuosis during radiotherapy is thus highly relevant.

## Results

### Ionizing radiation-induced immune protection

**Fig. 1:**
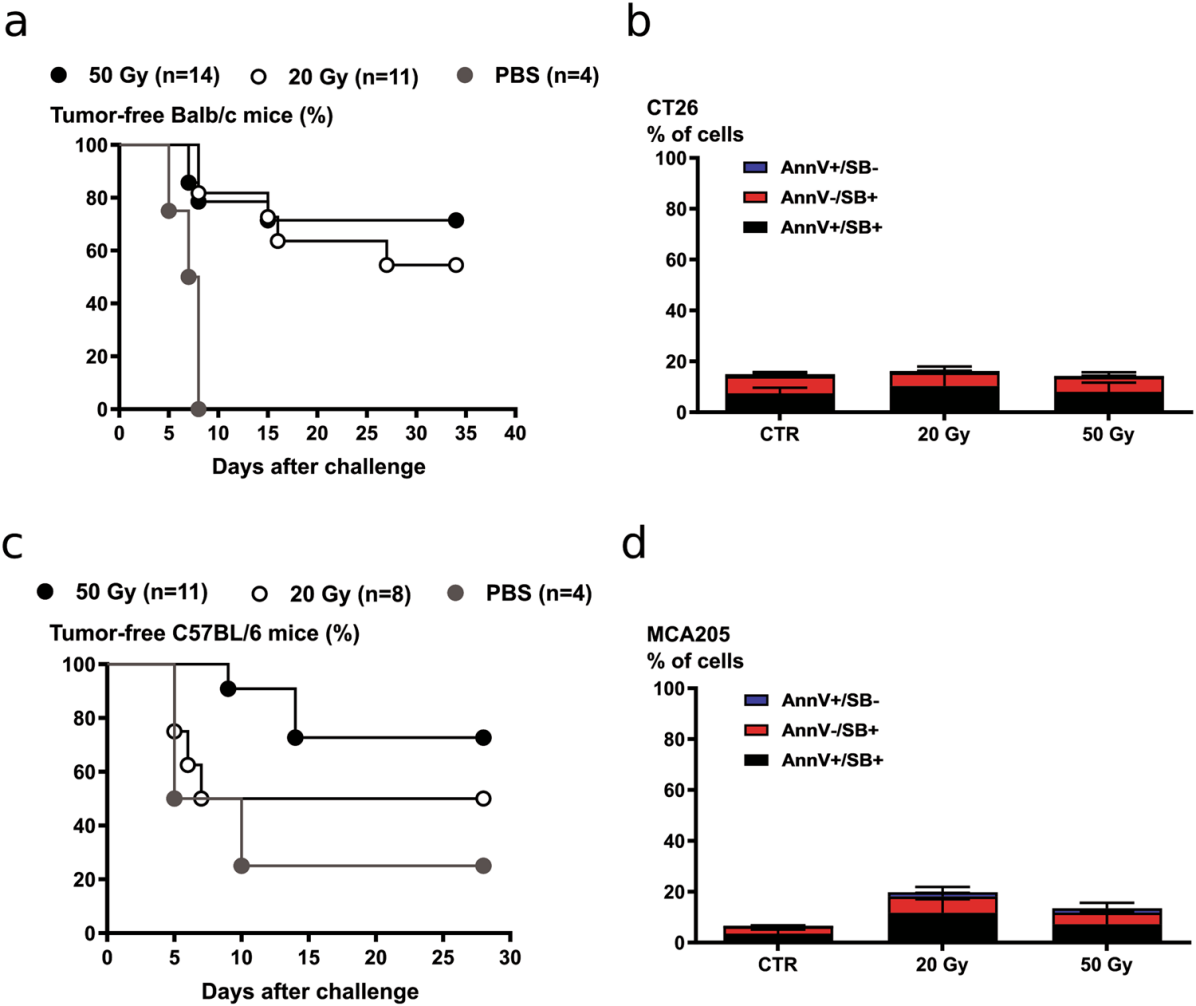
Prophylactic vaccination with high-dose irradiated cells (20 or 50 Gy) induce immune protection in mice. CT26 and MCA205 cells were irradiated with 20 or 50 Gy the day before the vaccination. PBS or cells irradiated with 20 and 50 Gy were injected subcutaneously in Balb/c. After 7 days, the mice were injected on the opposite flank with living cells. Tumor growth was monitored every 2 days up to 35 days post-challenge. Percentage of tumor-free mice after vaccination with irradiated CT26 cells and MCA205 cells are shown in **a** and **c**, respectively. The extent of cell death measured as Annexin V positivity (AnnV), Sytox Blue (SB) positivity, and a combination of the two was minimal at the time of injection and is shown in **b** (CT26 cells) and in **d** (MCA205 cells).

We tested whether as previously suggested^11,16^, a high-dose IR would be immunogenic, as opposed to a low dose. We performed prophylactic vaccination experiments with two cell lines commonly used in immunogenicity studies, the colon cancer cells CT26 and the fibrosarcoma cells MCA205. Cells were irradiated with a high dose (50 Gy), and with a lower dose IR (20 Gy) before subcutaneous injection into the flank of BALB/c or C57BL/6 mice. Although the extent of cell death was minimal at the time of injection (around 20% Sytox Blue positive Fig. 1b, d), the irradiated cells did not generate tumors at the vaccination site. Cells irradiated with doses lower than 20 Gy continued to grow in vivo, generating tumors on the vaccinated site (data not shown), thus invalidating the experiments performed with lower doses^24^. Cells irradiated with either 20 or 50 Gy were efficient in inducing an immune protection (Fig. 1a, c), and no statistical difference was observed between the two IR doses, in opposition with the abovementioned hypothesis.

### Induction of cell death by single-dose ionizing radiation in cell lines with altered cell death pathways

**Fig. 2:**
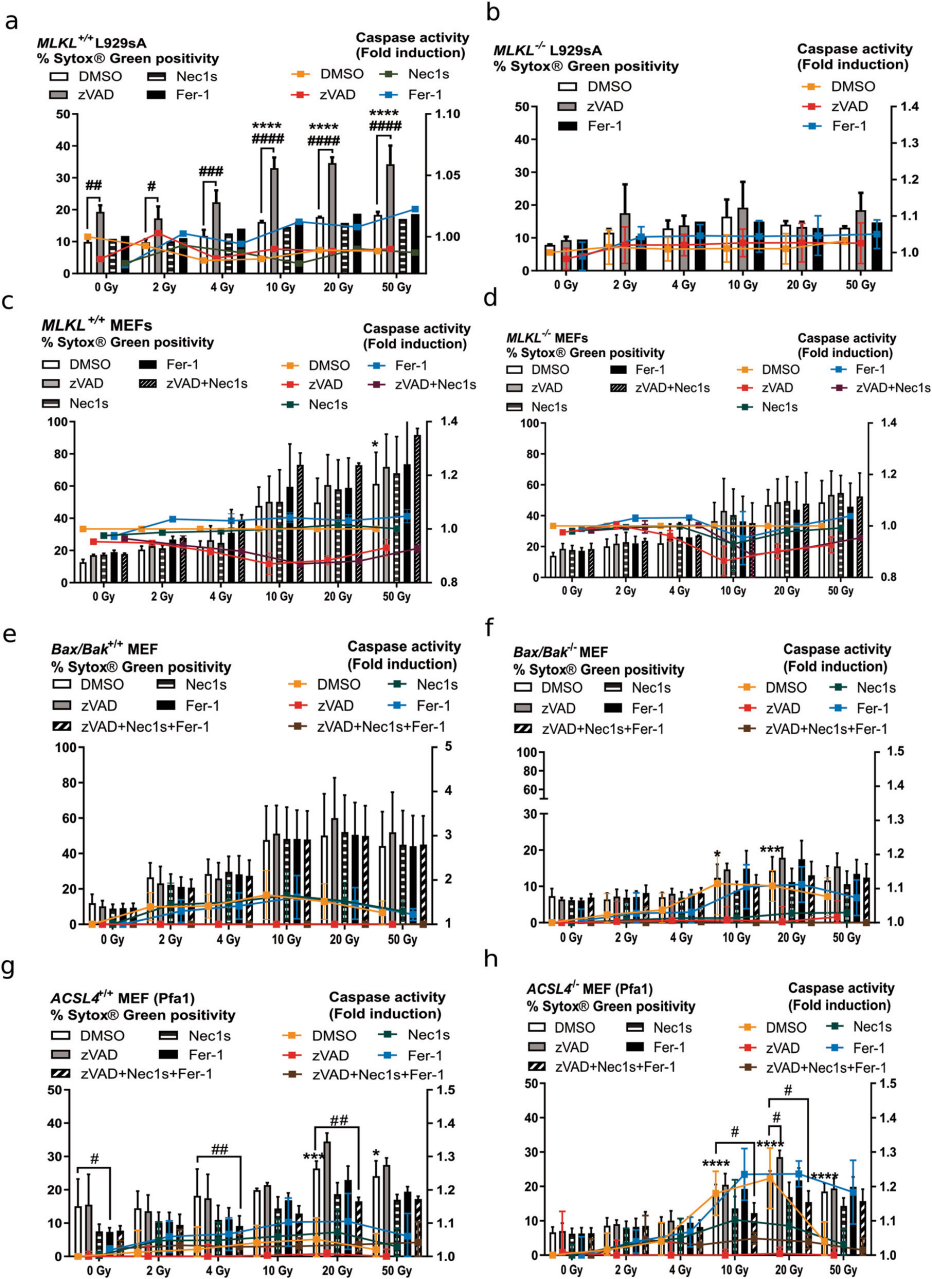
Cell death induction by single-dose ionizing radiation in cells with altered cell death pathways. **a–d** Cell death and caspase activity were measured 72 h after IR at the indicated doses by Sytox Green and Ac-DEVD-amc fluorescence in *MLKL*^*+/+*^ L929sA (**a**), *MLKL*^*−/−*^ L929sA (**b**), *MLKL*^*+/+*^ MEF (**c**), *MLKL*^*−/−*^ MEF cells (**d**), *Bax/Bak*^+/+^ MEF (**e**), and *Bax/Bak*^*−/−*^ MEF (**f**), *ACSL4*^*+/+*^ Pfa1 (**g**), and *ACSL4*^*−/−*^ Pfa1 (**h**). When indicated, the cells were pre-treated for 1 h with zVAD-fmk, Nec1s or Fer-1, or a combination thereof. Histogram bars show Sytox Green positivity and lines indicate caspase activity (fold induction). Means ± SEM are shown (*n* = 2–3). A two-way ANOVA was performed with a Tukey’s multiple comparisons test. Asterisk (*) shows the comparison to 0 Gy DMSO. Hash (#) shows the comparison to the DMSO treatment for the respective irradiation dose. *,^#^*p* ≤ 0.05, ^##^*p* ≤ 0.01, ***, ^###^*p* ≤ 0.001, ****,^####^*p* ≤ 0.0001.

One study reported the induction of necroptosis following radiation^13^. To evaluate whether in our experimental setup irradiated cells could undergo a necroptotic cell death modality, we used necroptosis proficient *MLKL*^*+/+*^ L929sA and *MLKL*^*+/+*^ MEFs as well as their deficient counterparts (*MLKL*^*−/−*^ L929sA and *MLKL*^*−/−*^ MEFs). L929sA cells were resistant to IR and did not undergo a significant increase in cell death as measured by Sytox Green uptake at any tested IR dose (Fig. 2a, b). We also did not find caspase activation as measured by DEVDase activity (Fig. 1a, b, right axes). Cell death increased when *MLKL*^*+/+*^ L929sA were exposed to IR doses in the presence of zVAD-fmk and was completely absent in *MLKL*^*−/−*^ L929sA (Fig. 2b), suggesting that it was necroptosis (Fig. 2a). Treatment with caspase inhibitor alone resulted in low levels of cell death, in line with a previous report^25^.

Both *MLKL*^*+/+*^ and *MLKL*^*−/−*^ MEF cells were sensitive to IR-induced cell death, especially at doses of 10 Gy and more (Fig. 2c, d). To investigate the cell death modality induced, we used zVAD-fmk (apoptosis), Nec1s (necroptosis), and Fer-1 (ferroptosis). Nec1s is an inhibitor of RIPK1 kinase affecting both RIPK1 kinase-dependent apoptosis and necroptosis^26^. To document a possible switch from apoptosis to necroptosis (or vice versa), we also included a combination with both zVAD-fmk and Nec1s (Fig. 2c, d). Caspase activation was not detected in these cell lines (Fig. 2c, d, right axes). The absence of protection by Nec1s on cell death in MEF suggests that RIPK1 is not implicated. We also used *RIPK1*^*−/−*^ MEF cells stably reconstituted with RIPK1-venus to visualize RIPK1 complex formation, quantified by the number of spots per area of cytoplasm with high-content imaging (Fig. S1). IR effectively induced cell death in these cells (dose-dependent decrease of the number of cells and increase in PI^+^ cells). No increase in the formation of RIPK1-venus dots was observed, while it clearly was in the necroptotic control (hTNF + Taki + zVAD-fmk). These results show that a wide range of IR doses did not induce necroptosis neither in L929sA nor in MEF cells. Only in the presence of zVAD-fmk, L929sA underwent necroptosis, induced by increasing IR doses, an artificial situation of caspase-8-inhibition. To investigate the contribution of intrinsic apoptosis in IR-induced cell death we used *Bax/Bak*^−/−^ and *Bax/Bak*^*+/+*^ MEF cells (Fig. 2e, f and Fig. S4b). Irradiated MEF cells deficient for Bax and Bak were resistant to cell death, suggesting their involvement in IR-induced cell death. *ACSL4*^*−/−*^ and *ACSL4*^*+/+*^ MEF cells (called Pfa1) were used to determine the contribution of ferroptosis in IR-induced cell death. The enzyme Acyl-CoA Synthetase Long-Chain Family Member 4 (Acsl4) catalyzes the conversion of long-chain fatty acids to their active form and is implicated in the synthesis of unsaturated long-chain fatty acids that are target for lipid peroxidation during ferroptosis. Cells lacking ACLS4 are resistant to ferroptotic cell death^27^. We did not observe a difference in the sensitivity to IR-induced cell death between these two MEF cell lines (Fig. 2g, h). These results prompted us to extend our study to investigate the effect of IR doses on a wider set of cancer cell lines.

### Single dose and fractionated IR-induced cell death in cancer cell lines

**Fig. 3:**
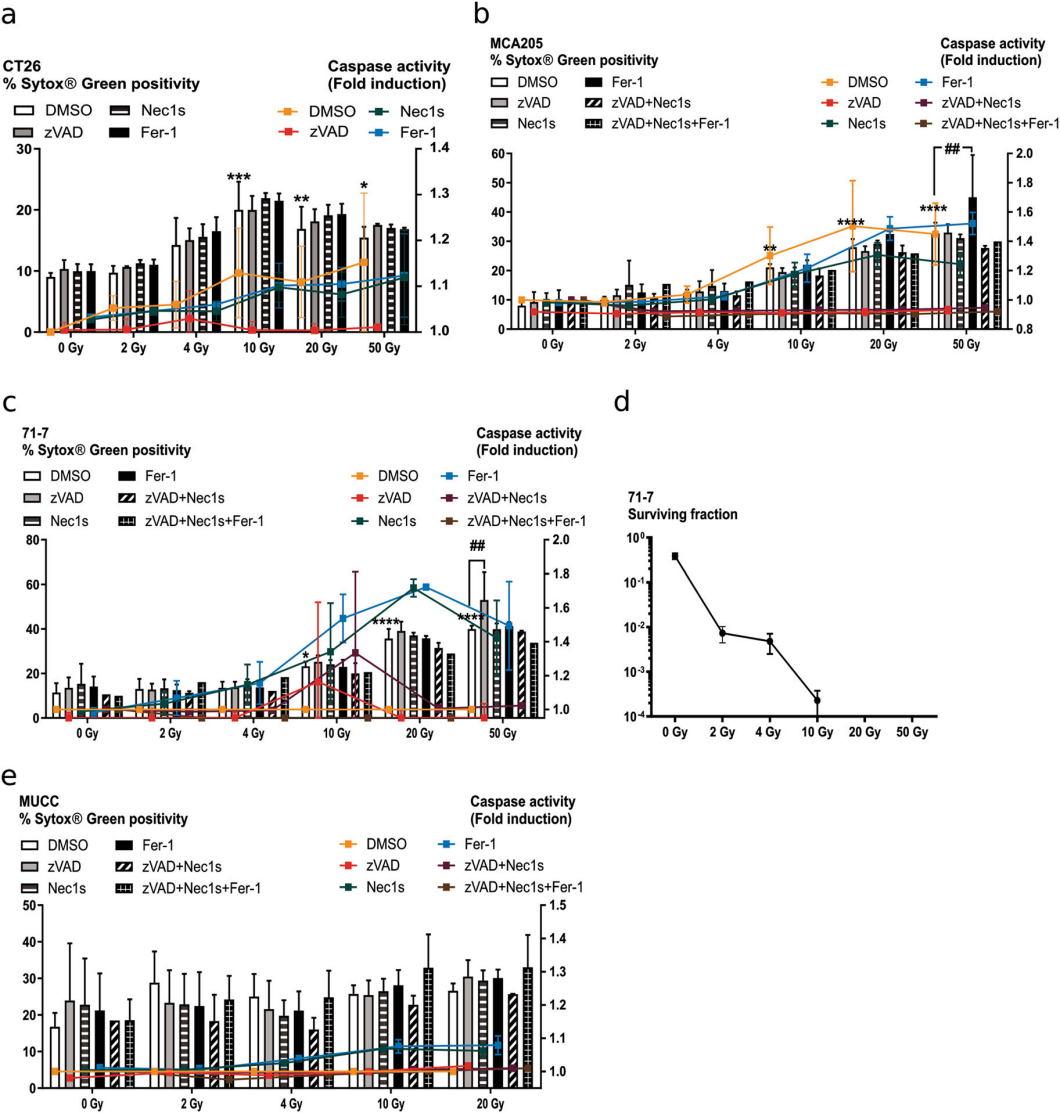
Cell death induction by single-dose ionizing radiation. **a–c**, **e** Cell death and caspase activity were measured 72 h after IR at the indicated doses by Sytox Green and Ac-DEVD-amc fluorescence in CT26 (**a**), MCA205 (**b**), 71-7 (**c**), and MUCC (**e**). When indicated, the cells were pre-treated with zVAD-fmk, Nec1s or Fer-1, or a combination thereof. Histogram bars reflect Sytox Green intensity and lines describe caspase activity (fold induction). **d** Clonogenicity assay performed on 71-7 cells at different IR doses. Means ± SEM are shown (*n* = 2–3). A two-way ANOVA was performed with a Tukey’s multiple comparisons test. Asterisk (*) shows the comparison to 0 Gy DMSO. *p ≤ 0.05, **,^##^*p* ≤ 0.01, ***p ≤ 0.001, ****p ≤ 0.0001.

We tested the effect of increasing IR doses on four cancer cell lines from different origins: CT26 colon carcinoma, MCA205 fibrosarcoma, 71-7 *Kras*^*G12D*^*;p53*^*−/−*^ lung adenocarcinoma, and the Murine Uterine Cervix Cancer (MUCC) cells. CT26, MCA205, and 71-7 showed a significant increase in IR-induced cell death from a dose of 10 Gy on (Fig. 3a–c), while the MUCC cells were not sensitive to IR-induced cell death at any of the IR doses tested (Fig. 3e). No inhibition of cell death was observed when pre-treated with either zVAD-fmk, Nec1s or Fer-1, or a combination thereof. We observed an IR dose-dependent increase in caspase activity in these cell lines. While zVAD-fmk could block the activity of caspases, no impact on cell death by plasma membrane permeabilization was observed, suggesting that the cells may continue to die by another cell death modality, different from necroptosis and bona fide ferroptosis. These results are in line with a recent study showing that zVAD-fmk could not protect against IR-induced cell death^28^. A clonogenic assay performed on 71-7 cells showed a complete cytostatic effect of IR at high doses (Fig. 3d), suggesting that cells lost their capacity to grow. We also performed a series of clonogenic assays with these four cancer cell lines and the same cell death inhibitors (Fig. S2a–d). While Nec1s, zVAD, and Fer-1 could rescue cell death induced by mTNF (necroptosis), staurosporine (apoptosis), and ML162 (ferroptosis), respectively (Fig. S2e), they did not rescue irradiated cells, in line with our results that other cellular stress and cell death modalities may be implicated.

Fractionated IR regimens are commonly used in the clinic and we tested whether repeated irradiation on the cells would induce higher levels of cell death and whether apoptosis, necroptosis, or ferroptosis would occur. Cell death slightly increased in *MLKL*^*+/+*^ and *MLKL*^*−/−*^ L929sA after fractionated IR compared to single doses (Fig. S3a, b). CT26, 71-7, and MUCC cells were more sensitive to fractionated than to single high IR dose (Fig. S3) and died significantly after being irradiated on three consecutive days with small doses such as 2 Gy (for CT26 and MUCC) and 3.3 Gy (71-7 cells) (Fig. S3c–e). As for single-dose IR, zVAD-fmk, Nec1s, or Fer-1 could not prevent the cell death induced, although zVAD-fmk could block caspase activity.

### Ionizing radiation induces features of mitotic catastrophe

**Fig. 4:**
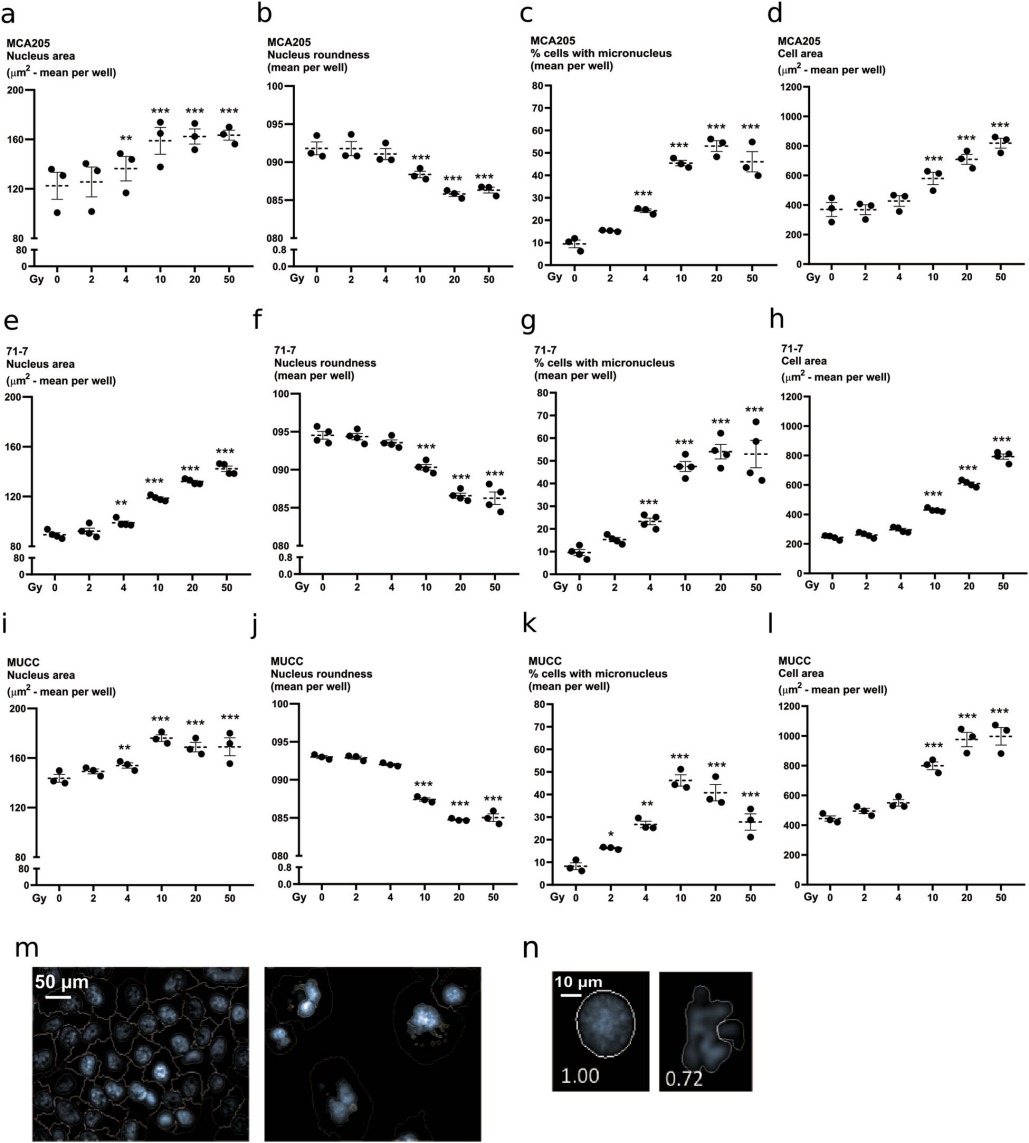
Induction of multinucleation and micronucleation by ionizing radiation. Nuclear morphology was quantified through high-content imaging in MCA205 (**a–d**), 71-7 (**e–h**), MUCC (**i–l**) cells, 48 h after irradiation. Cells were stained with Hoechst and propidium iodide after permeabilization to allow segmentation of the nucleus and cytoplasm, respectively. Nuclear area was measured in MCA205 (**a**), 71-7 (**e**), and MUCC (**i**). The roundness of the nucleus was also assessed after irradiation in MCA205 (**b**), 71-7 (**f**), and MUCC (**j**). Percentage of cells with micronuclei is shown for MCA205 (**c**), 71-7 (**g**), and MUCC (**k**) cells. The area of the cells was also measured following irradiation in MCA205 (**d**), 71-7 (**h**), and MUCC (**l**) cells. **m** Representative picture of micronuclei segmentation. **n** Representative picture of segmentation mask for the assessment of the nuclear shape.

Mitotic catastrophe is a cellular response that senses mitotic failure and drives cells to an irreversible antiproliferative fate leading to cell death or senescence^29^. As such, mitotic failure does not constitute a pure executioner cell death pathway but rather a cellular stress response following a mitotic defect as a consequence of IR. We sought to determine whether features of mitotic catastrophe would be present to a similar extent across all the IR doses examined. Following DNA damage, cells can fail to complete their mitotic cycle and this results in the presence of multiple nuclei and micronuclei. Nuclear morphology (area and roundness) and micronuclei formation were assessed by high-content imaging in irradiated MCA205, 71-7, and MUCC cells (Fig. 4a, b, e, f, i, j). Presence of micronuclei, irregular nuclear shape, and area increased with increasing IR doses (Fig. 4c, g, k). Cell size also increased in an IR dose-dependent way (Fig. 4d, h, l). These features were independent of p53 expression (p53 is expressed in MCA205, but not in MUCC and 71-7, Fig. S1). Together, these results show that increasing IR doses induce features of mitotic catastrophe, such as micronucleation, altered nuclear morphology, and multinuclei formation in three different cancer cell lines.

### IR-induced senescence

**Fig. 5:**
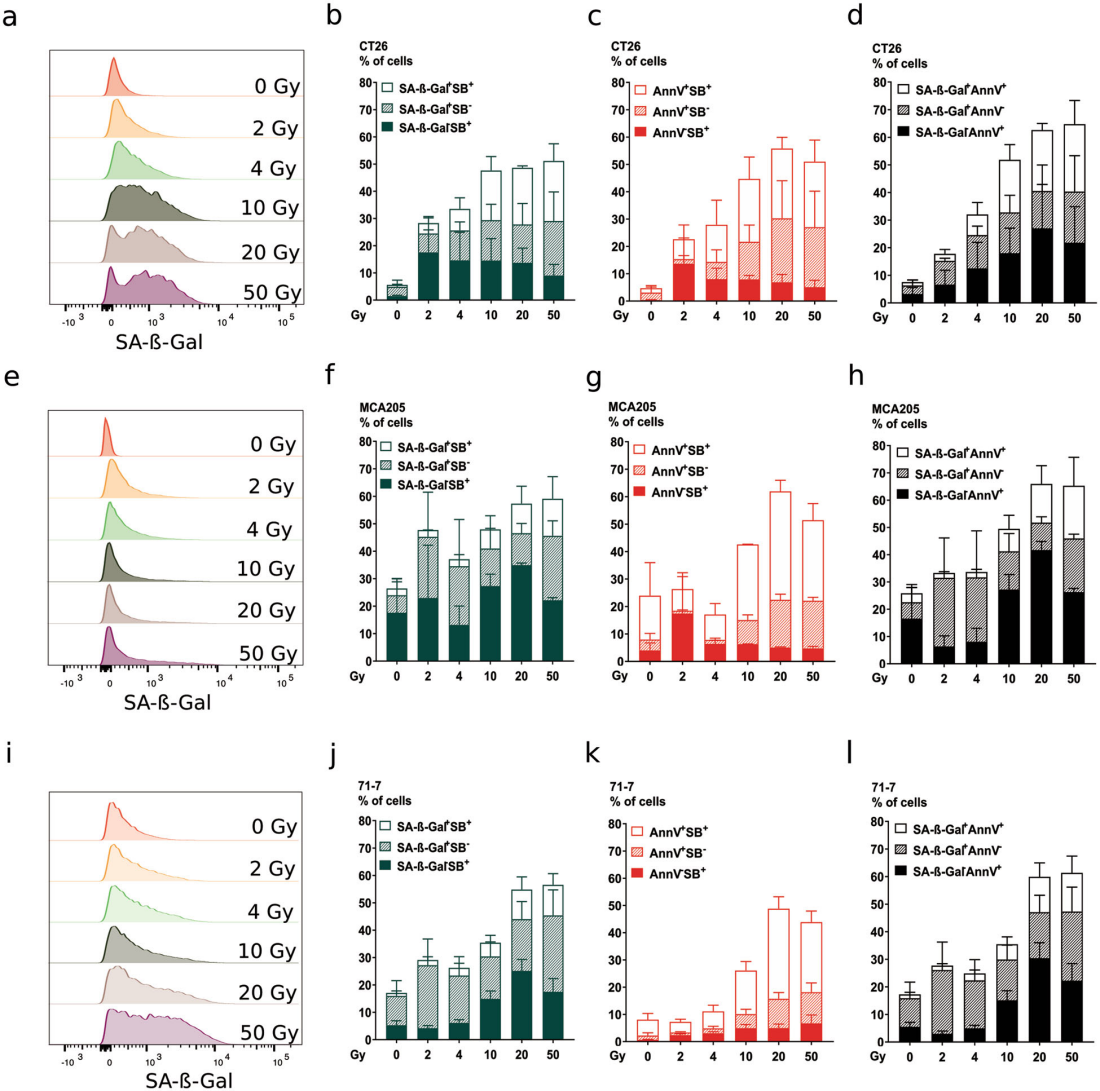
Ionizing radiation induces senescence. Senescence induction was measured 72 h following irradiation at the indicated doses by flow cytometry with C12FDG, a β-galactosidase substrate emitting fluorescence upon its cleavage. Co-staining with Annexin V-APC and Sytox Blue was performed to indicate apoptosis and membrane permeabilization, respectively, in CT26 (**a–d**), MCA205 (**e–h**), and 71-7 (**i–l**) cells. Representative flow cytometry histograms are shown for C12FDG (SA-β-Gal) staining in CT26 (**a**), MCA205 (**e**), and 71-7 (**i**). Percentage of cells positive for C12FDG (SA-β-Gal) and/or Sytox Blue is shown for CT26 (**b**), MCA205 (**f**), and 71-7 (**j**) cells. Percentage of cells positive for Annexin V-APC and/or Sytox Blue is shown for CT26 (**c**), MCA205 (**g**), and 71-7 (**k**). Percentage of cells positive for C12FDG (SA-β-Gal) and/or Annexin V-APC is shown for CT26 (**d**), MCA205 (**h**), and 71-7 (**l**). Means ± SEM are shown (*n* = 2). SA-β-Gal senescence-associated β-Galactosidase.

As mentioned above, mitotic catastrophe lead to cell death or senescence. The flat and enlarged morphology of cells following high-dose IR suggested a state of senescence. We performed a flow cytometry staining with a fluorescent β-galactosidase substrate, Annexin V and Sytox Blue, markers for phosphatidylserine exposure and membrane permeabilization, respectively. Flow cytometry histograms (Fig. 5a, e, i) show an IR dose-dependent increase in β-galactosidase activity for both CT26 and 71-7, while the increase was milder for MCA205. This is also reflected in the histograms showing the quantification of positive cells for β-galactosidase (Fig. 5b, f, j and d, h, l). We observed a dose-dependent increase of cells positive for both β-galactosidase and for the apoptotic marker Annexin V (Fig. 5d, h, l), suggesting that part of the senescent cells may eventually undergo apoptosis. These three cell lines showed caspase activity following IR (Fig. 3a–c), and here we also found an IR dose-dependent increase in apoptotic cells (Annexin V^+^/ Sytox Blue^−^) (Fig. 5c, g, k).

### Ionizing radiation-induced lipid peroxidation and iron-dependent cell death

**Fig. 6:**
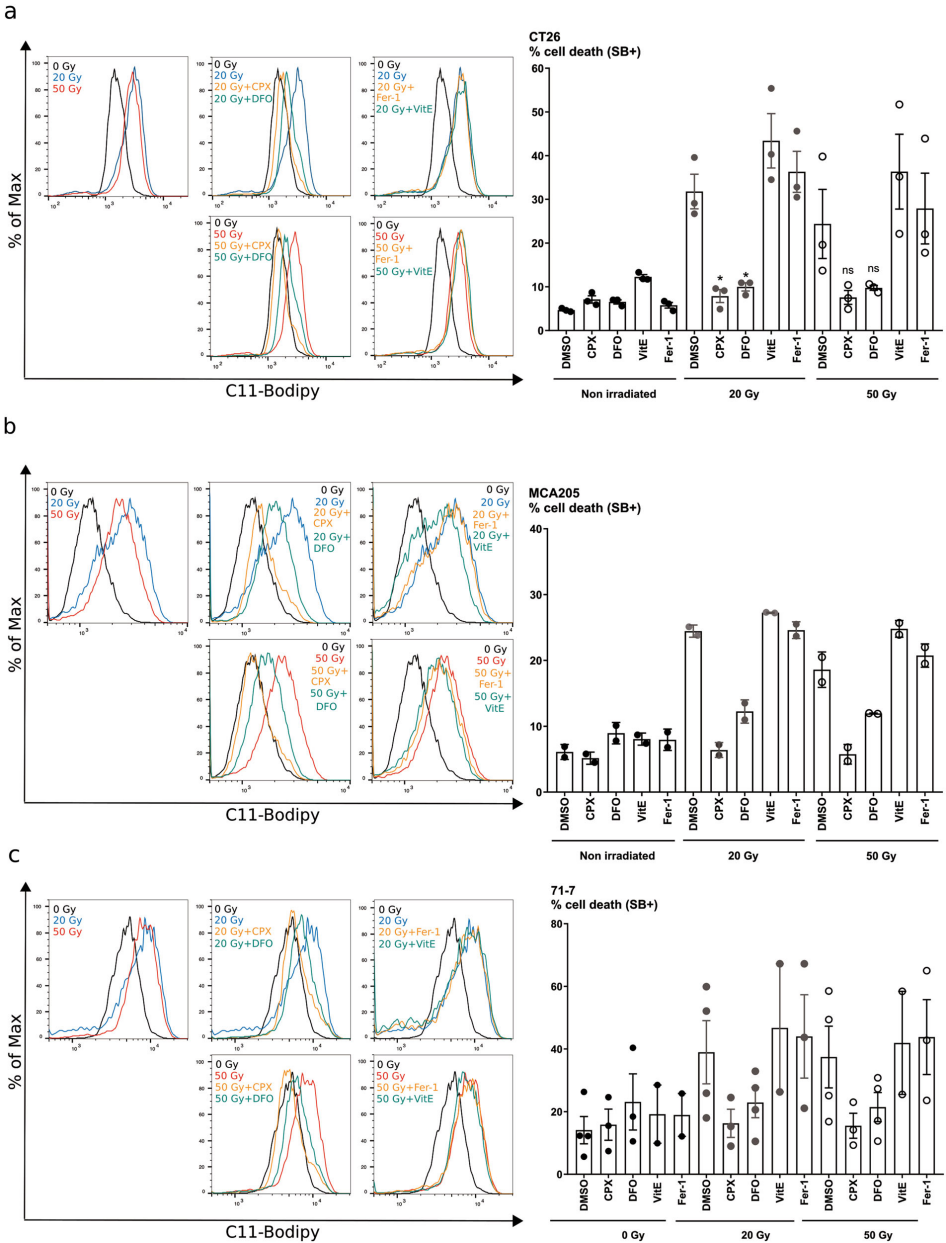
Ionizing radiation induces lipid peroxidation and an iron-dependent type of cell death. **a–c** Measurement of lipid peroxidation using C11-BODIPY probe and cell death using Sytox Blue in CT26 (**a**), MCA205 (**b**), and 71-7 (**c**) cells 24 h after irradiation with 20 or 50 Gy. Increase in lipid peroxidation can be seen in the first left quadrant by the shift of C11-BODIPY fluorescence measured after 20 Gy (blue) or 50 Gy (red) X-rays. When indicated, the cells were pre-treated with iron chelators CPX and DFO, or with antioxidants vitamin E and Fer-1 1 h before irradiation. Quantification of cell death is shown as percentage for CT26 (**a**), MCA205 (**b**), and 71-7 (**c**). Means ± SEM are shown (*n* = 2–3). A two-way ANOVA was performed with a Tukey’s multiple comparisons test. Asterisks (*) show the comparison to 20 Gy Methuosis. *p ≤ 0.05, **p ≤ 0.01.

We then investigated whether several cell lines exhibited lipid peroxidation following irradiation. We used the lipid peroxidation sensor, C11-Bodipy (508/591), which undergoes a shift in fluorescence when oxidized. All cell lines tested (CT26, MCA205, 71-7) revealed an increase in lipid peroxidation 24 h after high-dose IR (20 and 50 Gy) (Fig. 6a–c). We compared the effect of lipophilic antioxidants (Fer-1, Vitamin E) or iron chelators (CPX, DFO) on lipid peroxidation as demonstrated in the context of ferroptosis^17,18,30^. Interestingly only iron chelators, but not lipophilic free-radical traps, were able to block IR-induced lipid peroxidation and cell death induced by 20 and 50 Gy irradiation, suggesting that cell death was iron-dependent.

### Ionizing radiation induces features of methuosis

**Fig. 7:**
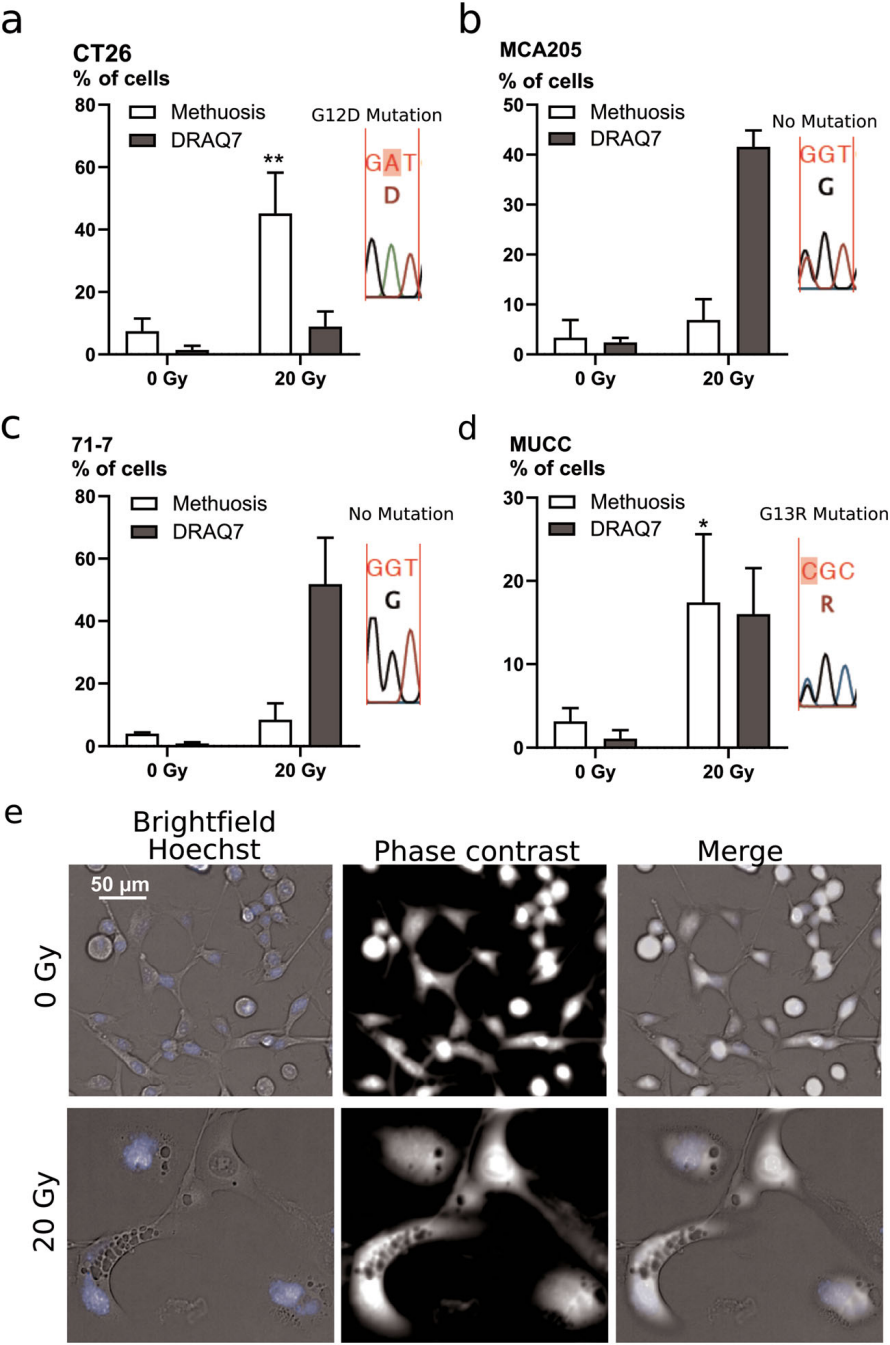
Ionizing radiation induces features of methuosis. Microscopic evaluation of the presence of phase-lucent vacuoles in CT26 (**a**), MCA205 (**b**), 71-7 (**c**), and MUCC (**d**) cells after 72 h irradiation with 20 Gy. An example of pictures for CT26 cells is shown in **e**. Means ± SEM are shown (*n* = 3). An unpaired *t*-test was performed for the methuosis data. Asterisks (*) show the comparison to 0 Gy. *p ≤ 0.05, **p ≤ 0.01.

By careful observation of the cellular morphology by microscopy, we detected the presence of large vacuoles visible in bright field and translucent in phase contrast. Such vacuoles are a feature of methuosis, a cell death modality triggered by Ras(G12V) activating mutation (H-Ras or K-Ras)^20,31^. We performed sequencing of K-Ras in our cell lines and found the G12D mutation in CT26 cells (Fig. 7a), according to the literature^32^. The activating mutation K-Ras(G13R) was found in the MUCC cells (Fig. 7d). No K-Ras mutations were found at position 12 or 13 in MCA205 and 71-7 cells (Fig. 7b, c). The latter result was unexpected, since 71-7 cells are derived from K-RasG12D, p53^−/−^ mouse tumors^33^. Significant increases in methuosis following irradiation were found in the two K-Ras mutated cells, CT26 and MUCC (Fig. 7a, d). These two cell lines show a low percentage of methuosis in non-irradiated condition, as could be expected from their active K-Ras mutation (Fig. 7a, e). In cell lines without K-Ras mutations (71-7 and MCA205), sporadic cells with methuotic features were also observed following irradiation (Fig. 7b, c, movies), suggesting that this process might also occur independently of K-Ras mutations (none of the cell lines had an H-Ras mutation at codon 61; data not shown). We followed the cells for 96 h after irradiation (Movies 1–4, Supplemental Material) and found morphological features of apoptosis (blebbing) in all the cell lines tested, presence of necrotic cells in CT26 and MCA205, and features of methuosis in CT26, MUCC, and 71-7 (Fig. S6a–d).

## Discussion

We show that in a panel of distinct cancer cell lines (colon, cervical, lung adenocarcinoma, fibrosarcoma), the cellular response to various regimens of irradiation was remarkably similar in a dose-dependent fashion. We observed a dose-dependent increase in caspase activity, mitotic catastrophe, senescence, and lipid peroxidation. We also found an increase in the presence of large translucent vacuoles that resemble methuosis in K-ras mutated cells.

Altogether, our observations are at odds with widely spread paradigms that low dose IR would eventually elicit apoptosis, while high dose would result in accidental necrosis^11,16^. We detected a dose-dependent increase in caspase activity and Annexin V+ cells after IR, and we could also observe apoptotic morphology (blebbing) by microscopy. However, while caspase activity could be blocked with the caspase inhibitor (zVAD-fmk), such treatment could not prevent cell death, suggesting that it might be overruled by another parallel cell death pathway. Caspase activation was shown to prevent release of type I interferon, which is involved in IR-induced immunogenicity^34,35^. Low levels of caspase activation following IR might allow for type I interferon release, explaining the immunogenicity observed. MEF cells lacking Bax and Bak were resistant to IR-induced cell death, suggesting that these pro-apoptotic proteins are important in this process. Earlier studies showed that when cells overexpressed Bax or Bak, treatment with zVAD-fmk could prevent caspase activation but was unable to inhibit cell death^36,37^, similar with our observation. Activation of Bax following irradiation seems to be essential to drive cell death through the mitochondria. In contrast, caspase activation seems to be optional for cell death to occur, as cells continue to die by other cellular stress mechanisms and cell death modalities.

Necroptosis was shown to be induced following IR with RIPK1 inhibitor Necrostatin-1 (Nec1) reducing the survival fraction cancer cells^13^. Here, necroptosis occurred only in the MLKL proficient L929sA, which was already shown upon addition of zVAD-fmk through autocrine TNF production^25^. None of the other cells line underwent necroptosis, even in the presence of zVAD-fmk, although the 71-7 and MEF cells exhibited an intact necroptotic pathway (expression of RIPK1 RIPK3 and MLKL). We conclude that in these cell lines, necroptosis is not the main cell death modality in response to IR.

ROS and free radicals produced following IR can react with lipids and generate lipid peroxides. Lipid peroxidation was shown to drive ferroptosis^38^ and cell death that can be inhibited by both iron chelators and antioxidants is termed ferroptosis^39^. Iron chelators can inhibit ferroptosis by preventing iron from donating electrons to oxygen, thereby preventing the formation of reactive oxygen species (ROS). Ferrostatin-1 and vitamin E are lipophilic inhibitors of ferroptosis which function as free-radical traps for peroxidized phospholipids^30,40^. Cell death and lipid peroxidation occurring following high-dose IR could only be blocked by iron chelators (DFO, CPX), but not by lipophilic free-radical traps, suggesting an alternative iron-dependent cell death modality different from bona fide ferroptosis.

Radiolysis of water generates hydroxyl radical (HO•) or hydrogen peroxide (H_2_O_2_). Cell death induced by IR resembles H_2_O_2_-induced cell death, which cannot be rescued by the antioxidant Fer-1, while it is by iron chelators^41^. A recent study found that IR triggers ferroptosis by inhibition of low levels of lipid peroxidation induced by 6 Gy radiation with the antioxidant Ferrostatin-1 (ref. ^42^). It is possible that Fer-1 could cope with the low levels of lipid peroxidation induced by 6 Gy, as opposed to the higher levels of lipid peroxidation seen with 20 or 50 Gy.

We also observed enhanced features of methuosis in cells expressing mutated K-Ras following IR. Cells that did not express K-Ras mutant showed only a minor presence of cells with vacuoles, suggesting that some of the vacuoles would be formed in a K-Ras-independent manner. Interestingly, the oncogene K-Ras is also known to induce senescence^43^. The relationship between senescence and methuosis has yet to be established, but from our microscopic analysis, we could see that part of the cells undergoing methuosis first presented a flat and enlarged morphology resembling senescence. Methuosis could be an eventual cell death modality outcome of senescence. Given the high prevalence of K-Ras mutation in different types of cancers, it would be interesting to further evaluate the importance of methuosis in the response to radiotherapy and whether this represents an immunogenic cell death modality or not.

**Fig. 8:**
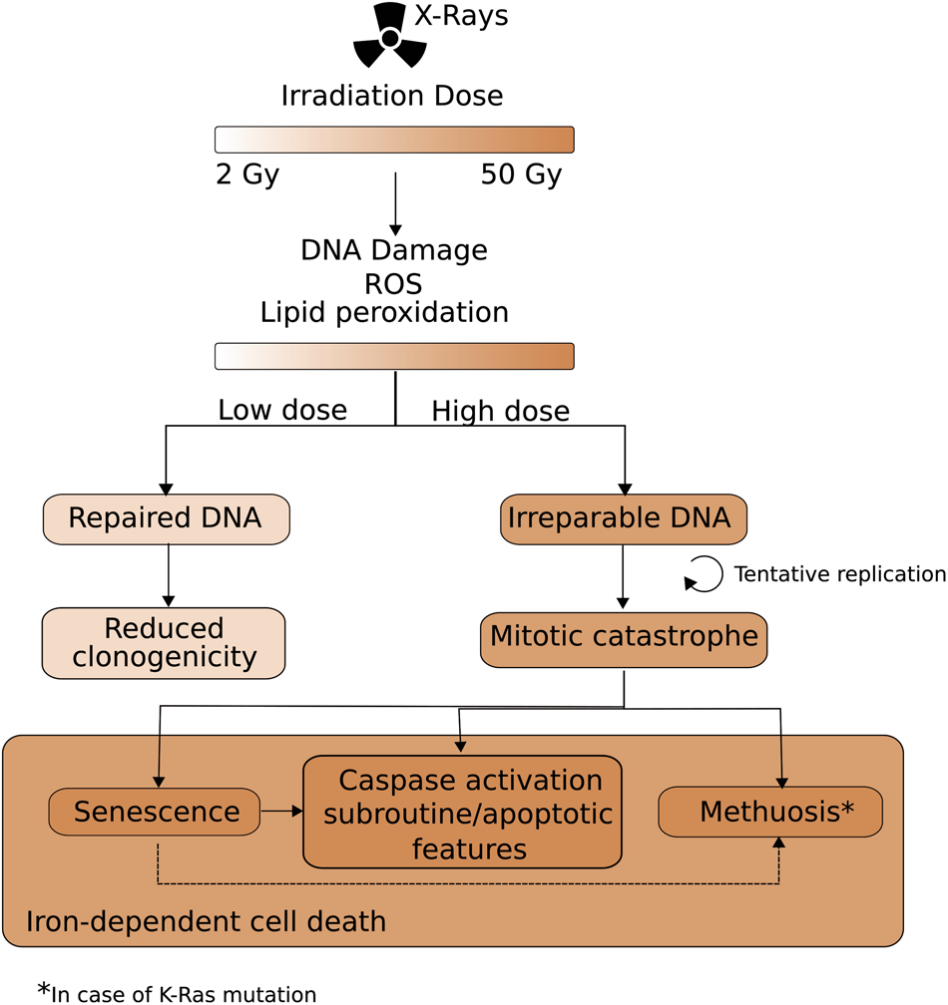
Cellular responses to ionizing radiation. Following X-ray irradiation with doses ranging from 2 to 50 Gy, there is a dose-dependent increase in ROS production, lipid peroxidation, and DNA damage. As a result, the DNA damage response is activated and will attempt to resolve the DNA breaks. In a case of low irradiation dose (<10 Gy), cells manage to repair their DNA, and still retain their capacity to proliferate. Cells with irreparable DNA that go into replication will be stuck in the mitotic cycle and undergo mitotic failure. Cells in a state of mitotic catastrophe will have several fates depending on the extent of the nuclear abnormalities and on the proteins expressed. Several outcomes of mitotic catastrophe seem to co-exist, such as senescence, methuosis, necrosis (membrane permeabilization), and activation of Bax, Bak, and caspases with presence of apoptotic features. A part of the senescent cells might become apoptotic (dotted arrow). K-Ras mutation can induce both senescence and methuosis; however, a link between the two cell states was never described. Here, we found that part of the large cells with flattened morphology also had methuotic vacuoles, which might indicate that methuosis could be an outcome of senescent cells (dotted arrow). Ultimately, the cells will die out of an iron-dependent type of cell death.

We propose a model in which upon increasing doses of IR, the cells are subject to an increasing amount of DNA damage and ROS^9^. ROS further impacts on the damage of the DNA and lipids, causing lipid peroxidation. The cell cycle is halted and the DDR is activated to repair the DNA, when possible. As evidenced by clonogenicity assays, when cells are irradiated with doses lower than 10 Gy, most of the DNA breaks can be repaired and the cells can resume their cell cycle, divide, and stay alive. However, with doses higher than 10 Gy, several DSB cannot be repaired. The next division round leads to mitotic catastrophe, and cells exhibit multiple nuclei and micronuclei. Cells in mitotic catastrophe can either die by apoptosis or necrosis, or become senescent^44^. Bax and Bak are implicated in IR-induced apoptosis, while blocking caspase activation reveals necrotic cell death different from necroptosis and bona fide ferroptosis. Senescent cells could also proceed to undergo apoptosis or unregulated necrosis^45^. Our findings demonstrate that neither RIPK1-dependent cell death nor bona fide ferroptosis occurs. Methuosis could be an outcome in K-Ras-mutated cells. Ultimately, cells undergo an iron-dependent necrosis associated with lipid peroxidation that cannot be blocked by lipophilic free-radical traps, but only by iron chelators (Fig. 8).

## Materials and methods

### Cell lines and reagents

MCA205 and 71-7 cells were cultured in RPMI supplemented with 10% FCS, 2 mM l-glutamine, 0.4 mM sodium pyruvate. MUCC cells were cultured in the same medium, but with a 20% FCS concentration. CT26, *MLKL*^*+/+*^ L929sAhFas (L929sA cells transfected with hFas)^46^ (later referred to as L929sA cells), *MLKL*^*−/−*^ L929sA (generated by Crispr/Cas9, by Jolien Bridelance), *MLKL*^*+/+*^ MEF, *MLKL*^*−/−*^ MEF, and *RIPK1-venus* MEF (generated by Maria Ladik) were cultured in Dulbecco’s modified Eagle’s medium supplemented with 10% FCS, 2 mM l-glutamine, 0.4 mM sodium pyruvate. All cells were maintained at 37 °C under 5% CO_2_ atmosphere. MCA205 were obtained from Guido Kroemer (Centre de Recherche des Cordeliers, Paris, France). 71-7 cells were obtained from Eileen White (Rutgers Cancer Institute of New Jersey, New Jersey, USA). MUCC cells were obtained from Katrien Vandecasteele (Ghent University Hospital, Radiotherapy Department, Ghent, Belgium). ACSL4^−/−^ and ACSL4^+/+^ MEF cells (called Pfa1) were a kind gift from Marcus Conrad (Helmholtz Zentrum München, Neuherberg, Germany). Cell lines were regularly tested for mycoplasma.

zVAD-fmk (Bachem, Bubendorf, Switzerland) was used at 20 μM; Necrostatin 1s (Nec1s) (synthesized by the Laboratory of Medicinal Chemistry, University of Antwerp) was used at 10 μM. Antioxidant Ferrostatin-1 (Fer-1) (Xcess Biosciences) was used at 10 μM; vitamin E (α-tocopherol) (Sigma-Aldrich) was used at 100 μM. The iron chelators DFO (Sigma-Aldrich) and Ciclopirox olamine (CPX; Sigma-Aldrich) were used at 100 and 10 μM, respectively. Human TNF was used at 600 U/mL and Tak1 inhibitor (Taki; Analyticon Discovery) at 1 μM. For flow cytometry, Annexin V-APC (BD Biosciences) was used at a 1:400 dilution and Sytox Blue (Molecular Probes) was used at 1.3 μM. For Fluostar cell death assay, Sytox Green (Molecular Probes) was used at 1 μM.

For western blot, the following antibodies were used: anti-RIPK1, anti-RIPK3 (Sigma-Aldrich, R4277), anti-MLKL (Millipore, MABC604), anti-caspase 3 (Cell Signaling Technology, 9662), anti-caspase-8 (Abnova, MAB3429), anti p53 (Cell Signaling Technology, 2524), anti-b-tubulin-HRP (Abcam, ab21058), anti-Bax (Upstate Biotechnology, 06-499), anti-Bak (Millipore, 06–536), anti-ACSL4 (Santa Cruz Biotechnology, sc-271800).

### X-ray irradiation of cells

Cells were irradiated using a clinical 6 MV X-ray beam produced by a linear accelerator Elekta Synergy® (Elekta Crawley) at the Radiation Oncology Department (University Hospital Ghent, Ghent University). Source-to-surface distance was 1 m; a 1.5 cm Perspex® plate was used to compensate the build-up effect.

### Prophylactic vaccination with irradiated cells

Female BALB/c (Charles River, France) or C57BL/6 (Janvier, France), 7- to 8-week-old were housed in a pathogen-free facility in the animal facility of the IRC-VIB Department. All animal study procedures were approved by the ethical committee of Ghent University. CT26 and MCA205 cells were irradiated with 20 or 50 Gy the day before the vaccination. Mice were vaccinated with 3 × 10^6^ irradiated CT26 subcutaneously on the left flank and were then challenged with 5 × 10^5^ live cells 7 days later on the opposite flank. Vaccination with irradiated MCA205 cells was performed with 3 × 10^5^ cells and the challenge with 3 × 10^4^ cells. Mice developing tumors at the vaccination site were not included in the experiment according to the immunogenic cell death guidelines^47^. The tumors were measured using calipers every alternate day. Mice were randomly assigned a cage and the outcome was assessed blindly.

### Analysis of cell death

Cell death was measured with a Fluostar Omega plate reader (BMG Labtech, Ortenberg, Germany) as previously described^48^. Cells were seeded in triplicate at 1000 cells per well in 170 μL of medium in 96-well plates. Eight hours later, the cells were pre-treated 30 min with cell death inhibitors zVAD-fmk, Nec1s, Fer-1 before being irradiated at the indicated doses. At 48 h post-irradiation, the cells were incubated for 1 h at 37 °C with 1 μM of Sytox Green (Thermo Fisher scientific) and 10 μM of Ac-DEVD-AMC (PeptaNova, GmbH, Sandhausen, Germany). Sytox green intensity and caspase activity were measured with a Fluostar Omega plate reader both after 48 and 72 h post-irradiation with ex/em filters of 485/520 nm for Sytox Green and 360/460 nm for Ac-DEVD-AMC. 0.1% Triton X-100 was used to permeabilize the cells and obtain the maximal cell death for each well. Cell death was calculated by subtracting the background fluorescence from Sytox Green intensity, divided by the maximal cell death subtracted from the background fluorescence.

### β-galactosidase staining for senescence

Murine cancer cell lines (71-7, CT26, and MCA205) were cultured to ~80% confluence in 12-well plates and subjected to X-ray irradiation at the doses mentioned. Control non-irradiated (NI) cell lines were cultured to equivalent confluence and handled in the same manner as irradiated cells and control non-senescent (NS) cells were freshly seeded the day before analysis at ~90% confluence. Cellular senescence was assessed according to Debacq-Chainiaux et al. (2009)^49^. Measurements were performed 72 h post-irradiation using flow cytometry. Bafilomycin A1 (Sigma, 100 nM) was added for 1 h to induce lysosomal alkalynization. This step was followed by 2 h incubation with 5-dodecanoylaminofluorescein di-β-d-galactopyranoside C12FDG, Thermo Fisher Scientific, 33 μM), a fluorogenic substrate for β-galactosidase activity (Ex 490/Em 514). Once inside the cell, the C12FDG substrate is cleaved by β-galactosidase producing a green fluorescent product measured with the LSRII (BD PharMingen). The results were analyzed with FlowJo 10.4 Software (Treestar, Ashland, OR).

### C11-Bodipy staining

Cells were seeded at 100,000 cells per well in 12-well plates. Once adherent, the cells were pre-treated with the lipophilic free-radical traps (Fer-1, Vit E) and iron chelators (DFO and CPX). The cells were then irradiated with 20 or 50 Gy. Twenty-four hours after irradiation, the cells were harvested, washed with phosphate-buffered saline (PBS), followed by staining with Bodipy^TM^ 581/591 C11 (Thermo Fisher Scientific, D3861) (2 μM final concentration) in PBS, at 37 °C in the dark for 10 min. Cells were then washed with PBS before being incubated with Sytox Blue (1.25 μL final concentration) in PBS. Samples were acquired with a BD LSRII flow cytometer (BD Biosciences) and results were analyzed with FlowJo 10.4 software.

### Clonogenicity assay

71-7 cells were irradiated at indicated doses. After 24 h, 500 cells were seeded per T25 flask. After 10 days, when colonies are formed, cells were fixed with 4% PFA for 10 min, washed twice with PBS, and subsequently stained with 0.01% crystal violet diluted in PBS for 1 h RT. Finally, the cells were washed and dried overnight before imaging with a Nikon microscope AZ100M. Colonies were counted with Fiji.

### Assessment of nuclear morphology

Nuclear morphology was assessed using an Operetta high-content imager (PerkinElmer). Five thousand cells were seeded in 96-well plates. Once adherent, the cells were irradiated at the indicated doses. Forty-eight hours post-irradiation, 3 μM Hoechst (Sigma-Aldrich and 1 μM propidium iodide (Sigma-Aldrich) were added to the cells before image acquisition with a ×20 objective. After acquisition, cells were fixed with 4% homemade Paraformaldehyde (PFA) for 10 min, and subjected again to the Operetta high-content imager. Nuclear area, roundness, and cell area were assessed with Colombus software (PerkinElmer). Segmentation masks were made with Hoechst for the nucleus and for the cytoplasm with propidium iodide. The experiment involved either three or four biological replicates and sub-sampling with 18 technical replicates as sampling units.

### Quantification of methuosis

The presence of phase-lucent vacuoles was assessed with an Opera High-Content Imager (PerkinElmer). Two thousand and five hundred cells were seeded in 96-well plates. The next day, cells were irradiated with 20 Gy. Cells were stained 72 h post-irradiation with 3 μM Hoechst and 0.6 μM DRAQ7 (Biostatus, Leicestershire, UK), before acquisition with a ×20 objective. The number of nuclei and DRAQ7-positive cells was calculated with segmentation masks using Columbus software (PerkinElmer). The number of cells with phase-lucent vacuoles was determined manually.

For the movies examining cellular morphologies, 2000 cells were seeded per well in 96-well plates. The next day, cells were irradiated with 20 Gy. Three hours after irradiation, 1 μM Sytox Green (Thermo Fisher Scientific) was added to the cells and the plates were placed in an Incucyte ZOOM platform, inside an incubator at 37 °C with 5% CO_2_. Four ×20 magnification images from different areas per well were captured at 1-h interval with phase contrast and green (800 ms exposure) channels for up to 4 days post-irradiation. Each condition was run in triplicates. Incucyte software’s processing definition was used to generate representative images.

### Statistical analysis of nuclear morphological data

A hierarchical generalized linear mixed model (HGLMM) as implemented in Genstat v19 (VSN International (2017), Genstat *for Windows* 19th Edition, VSN International, Hemel Hempstead, UK. Web page: Genstat.co.uk) was fitted to the data. Fixed terms included the cell-type, the irradiation dose and their interaction; random terms included the biological replicate, the plates nested within the replicate and the subsamples nested within the plate. In the case of cell area, nuclear area, nuclear roundness and MN+ cells, an HGLMM was fitted considering a normal distribution for both fixed and random effects. In the case of number of objects and number of micronuclei, a Poisson distribution of the fixed effects and a Gamma distribution of the random effects were considered. Significances of main and interaction effects were assessed by a WALD test for dropping fixed terms. *T* statistics were used to assess the significance of specific fixed effects estimated as differences (on the transformed scale) to a reference level (Gy = 0). Estimated mean values were obtained as predictions from the HGLMM, formed on the scale of the response variable.

## Supplementary information

Supplemental figure legends

Figure S1

Figure S2

Figure S3

Figure S4

Figure S5

Figure S6

Movie 1 CT26

Movie 2 MUCC

Movie 3 MCA205

Movie 4 71-7
