# Supplementary material for: Ionizing radiation results in a mixture of cellular outcomes including mitotic catastrophe, senescence, methuosis, and iron-dependent cell death
Source: Cell Death Dis. 2020 Nov 23;11(11):1003. doi: 10.1038/s41419-020-03209-y (PMC7684309; doi:10.1038/s41419-020-03209-y)
Supplement: Supplementary file 1 — Supplemental figure legends [file 41419_2020_3209_MOESM1_ESM.docx]

**SUPPLEMENTARY INFORMATION**

**Figure S1. IR does not induce RIPK1 complex formation**

High-content imaging for the quantification of cell death and RIPK1 aggregates in RIPK1-*venus* cells irradiated and stained with hoechst and propidium iodide. When indicated, cells were pretreated with Nec1s for 1h before irradiation. Representative pictures of non-treated cells, cell irradiated with 10 Gy, cells undergoing necroptosis (hTNF, Taki, zVAD-fmk) or not (hTNF, Taki, zVAD-fmk, Nec1s) are shown. Histograms show the number of cells per well, the percentage of PI positive cells and the number of spots/area of cytoplasm.

Means ± SEM are shown (n=2-3). A two-way ANOVA was performed with a Tukey’s multiple comparisons test.

**Figure S2 Survival of cancer cells following IR**

Surviving fractions of CT26 (**a**), MCA205 (**b**), 71-7 (**c**) and MUCC (**d**) are shown upon IR with 2 Gy, 4 Gy and 10 Gy, as assessed by clonogenic assay. When indicated, the cells were pretreated for 1h with zVAD-fmk, Nec1s or Fer-1, or a combination thereof. Inhibitors were added again after reseeding for the duration of the assay. Relative numbers of colonies are shown upon treatment of CT26 cells with mTNF, STS and ML126 alone or in combination with the inhibitors Nec-1s, zVAD, and Fer-1 respectively.

**Figure S3. Cell death induction by fractionated ionizing radiation**

(**a-e**) Cells were irradiated at the indicated doses on three consecutive days and cell death and caspase activity were measured 72h after the last IR by Sytox Green (Histogram bars) and Ac-DEVD-amc (lines) fluorescence in L929sA *MLKL^+/+^* (**a**), L929sA *MLKL^-/-^* (**b**), CT26 (**c**), 71-7 (**d**), MUCC cells (**e**). When indicated, the cells were pretreated with zVAD-fmk, Nec1s, Fer-1 or a combination thereof. Means ± SEM are shown (n=2). A two-way ANOVA was performed with a Tukey’s multiple comparisons test. Asterik (*) shows the comparison to 0 Gy DMSO. *p≤0.05, **p≤0.01, ***p≤0.001.

**Figure S4. Characterization of the cell lines used**

(**a**) Western blot showing the expression of RIPK1, RIPK3, MLKL, Caspase 3 and 8, p53 for all the cell lines used in this study. (**b**) Western blot showing the knock-out of Bax and Bak in MEF cells and the knock-out of ACSL4 in Pfa1 cells.

**Figure S5. ML162-induced lipid peroxidation.**

Increase in lipid peroxidation can be seen in the first left quadrant by the shift of C11-BODIPY (purple) upon ferroptosis inducer ML162 in CT26 cells. Pre-treatment with iron chelators CPX and DFO prevented lipid peroxidation as shown in the second quadrant (green and orange respectively).

**Figure S6. Cell death morphologies following IR**

Necrotic, apoptotic and methuotic morphological features observed by microscopy in CT26 (**a**), MUCC (**b**), MCA205 (**c**), 71-7 (**d**) cells. Images derived from movies 1, 2, 3, 4 respectively.

**Movie 1.** CT26 cells irradiated with 20 Gy and followed for 6 days

**Movie 2.** MUCC cells irradiated with 20 Gy and followed for 5 days

**Movie 3.** MCA205 cells irradiated with 20 Gy and followed for 6 days

**Movie 4.** 71-7 cells irradiated with 20 Gy and followed for 6 days
