# Supplementary figures and images for: Ionizing radiation results in a mixture of cellular outcomes including mitotic catastrophe, senescence, methuosis, and iron-dependent cell death
Source: Cell Death Dis. 2020 Nov 23;11(11):1003. doi: 10.1038/s41419-020-03209-y (PMC7684309; doi:10.1038/s41419-020-03209-y)

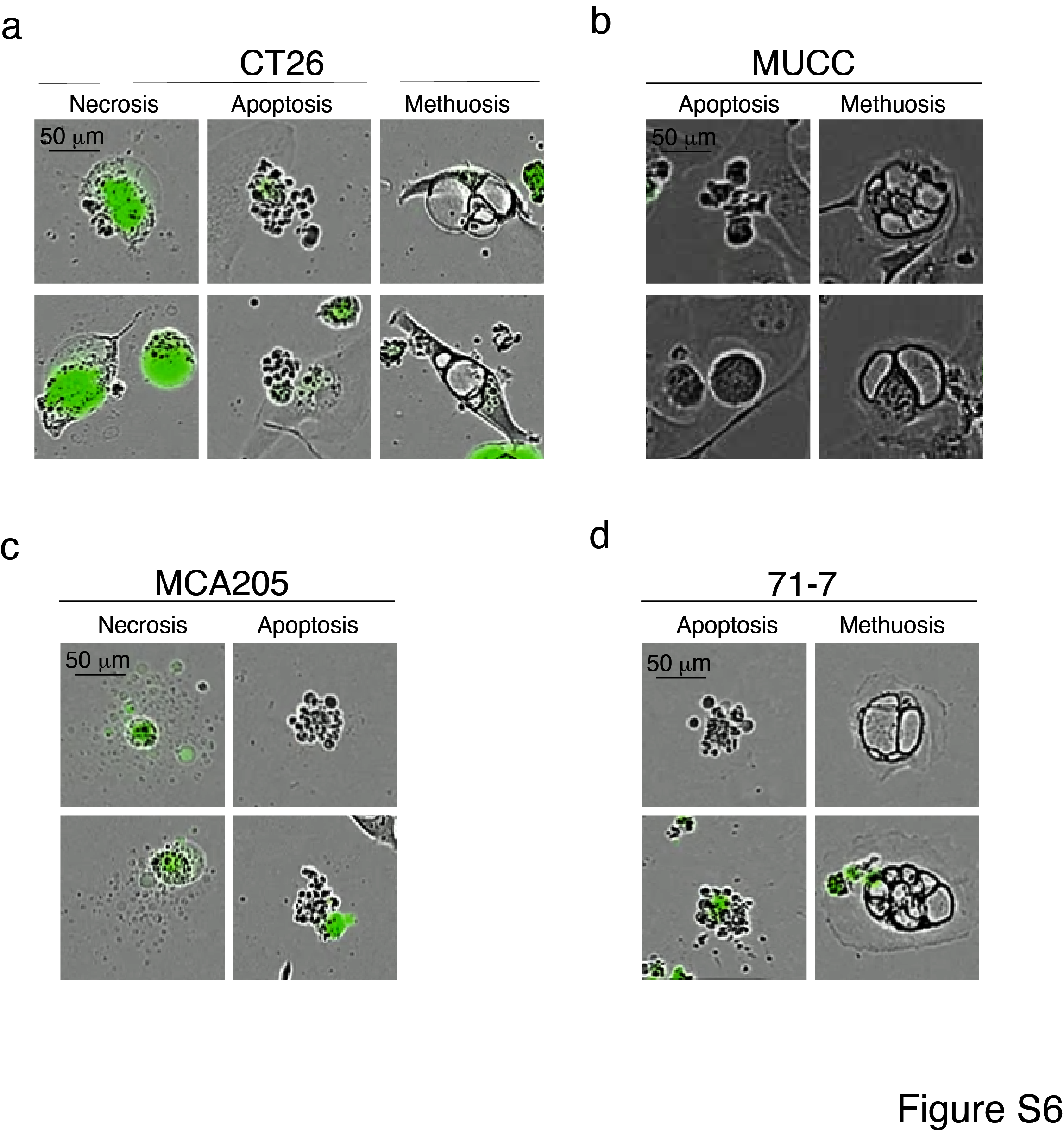

Supplement: Supplementary file 7 — Figure S6 [file 41419_2020_3209_MOESM7_ESM.tif]
